# Incidence, Patient-Directed Discharge, Readmission, and Mortality Among People Hospitalized With Injecting-Related Infection: A Population-Based Linkage Study

**DOI:** 10.1093/ofid/ofaf257

**Published:** 2025-04-29

**Authors:** Jeffrey Masters, Brendan Jacka, Marion Barault, David Goodman-Meza, Danielle Russell, Gail V Matthews, Gregory J Dore, Heather Valerio, Marianne Martinello

**Affiliations:** Kirby Institute, UNSW Sydney, Sydney, Australia; Kirby Institute, UNSW Sydney, Sydney, Australia; Kirby Institute, UNSW Sydney, Sydney, Australia; Kirby Institute, UNSW Sydney, Sydney, Australia; Kirby Institute, UNSW Sydney, Sydney, Australia; Kirby Institute, UNSW Sydney, Sydney, Australia; Kirby Institute, UNSW Sydney, Sydney, Australia; Kirby Institute, UNSW Sydney, Sydney, Australia; Kirby Institute, UNSW Sydney, Sydney, Australia

**Keywords:** infective endocarditis, injecting-related infections, opiate agonist therapy, patient-directed discharge, persons who inject drugs

## Abstract

**Background:**

Despite increasing hospitalization for injecting-related infection, there has been limited large-scale evaluation of in-hospital and post-discharge outcomes. This study aimed to characterize population-level injecting-related infection hospitalization and correlates associated with patient-directed discharge, readmission, and all-cause mortality among persons who inject drugs with hepatitis C in New South Wales, Australia, between 2001 and 2022.

**Methods:**

Hepatitis C notifications in New South Wales were linked to data involving hospitalizations, opioid agonist treatment, incarceration, and death registration. Hospitalizations among people who inject drugs with injecting-related infections were identified by *ICD-10* code algorithms. Incidence of patient-directed discharge, readmission, and mortality was calculated, and correlates associated with each outcome were assessed by extension of a Cox proportional hazards model for recurrent events.

**Results:**

In total, 18074 injecting-related infection hospitalizations were included among 9045 individuals, predominantly males (64%) with an average age of 41 years. The incidence was 47.2 per 100 person-years and increased over time. The proportion of hospitalizations ending in patient-directed discharge was 18% and was associated with stimulant use and incarceration, and lower in those with severe disease and opiate agonist therapy. The proportions of hospitalizations that were followed by 30-day and 1-year readmission were 25% and 61%, respectively, and had a strong association with patient-directed discharge. Mortality was 2% at 30 days and 15% at 1 year post-discharge.

**Conclusions:**

Patient-directed discharge was common among people admitted with injecting-related infections and was associated with readmission but not mortality. Person-centered models of care are necessary to address the health inequity experienced by people who inject drugs.

## Teaser

In this large-scale data linkage analysis that characterized injecting-related infections, the incidence increased, and patient-directed discharge was common, leading to high readmission rates. Other factors were explored to identify patients at highest risk for poor outcomes and target interventions.

## Graphical Abstract

**Graphical Abstract:**
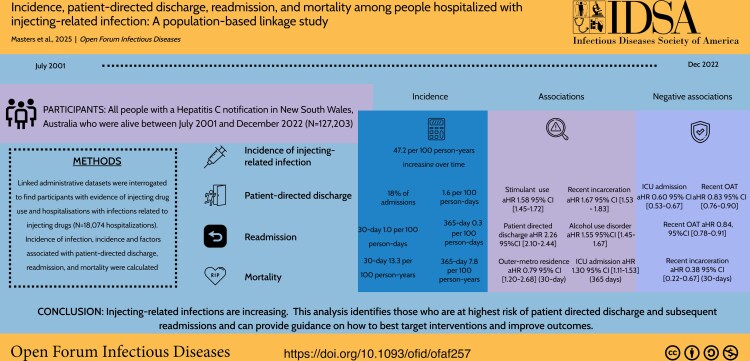
This graphical abstract is also available at Tidbit: https://tidbitapp.io/tidbits/incidence-patient-directed-discharge-readmission-and-mortality-among-people-hospitalized-with-injecting-related-infection-a-population-based-linkage-study?utm_campaign=tidbitlinkshare&amp;utm_source=ITP

For persons who inject drugs, a plethora of physical, social, and societal factors produces an environment that can lead to substantial drug-related harms. Bacterial and fungal infections acquired during drug injection (hereafter, injecting-related infections) are a frequent clinical complication [1–3]. Localized infections of the skin and soft tissues (eg, abscesses and cellulitis) are most common [3]. While less common, severe and/or systemic infections (eg, endocarditis, sepsis, osteomyelitis, and septic arthritis) have significant individual and health system impact, often requiring extended hospitalization (eg, 2–6 weeks) for management, including administration of parenteral antimicrobial therapy.

Intersecting social and structural factors in hospital settings affect persons who inject drugs, where experiences of stigma, discrimination, and inadequate pain relief and withdrawal management can precipitate patient-directed discharge [4]. Incomplete treatment, including antimicrobial therapy, can negatively affect patient health, increasing risk for hospital readmission and postdischarge mortality. Increasing numbers of hospitalizations for the treatment of injecting-related infections have been reported in several high-income countries [5–11], alongside additional expenditure for health care systems [6, 12]. Given the scale of injecting drug use globally (estimated 14.8 million people) and inadequate access to harm reduction in most countries (eg, needle-syringe programs, opiate agonist therapy [OAT], or supervised injecting facilities) [13, 14], the burden of injecting-related infections is expected to be considerable. While there is evidence of increasing occurrence of hospitalization for injecting-related infection, few studies have comprehensively examined in-hospital and postdischarge outcomes.

This study aimed to describe population-level trends in hospitalization for bacterial and fungal injecting-related infections and related outcomes among persons who inject drugs with a hepatitis C virus (HCV) notification in New South Wales (NSW), Australia, 2001–2022. Using administrative data linkage, this study reported the incidence of and factors associated with patient-directed discharge, hospital readmission, and all-cause mortality within 30 and 365 days of discharge.

## METHODS

### Data Sources

For context, NSW is the most populous state in Australia with a population of >8.2 million persons at the end of the study period, December 2022 [15]. The NSW hepatitis C data linkage project has been previously described [16]. In brief, this data source holds all HCV notifications made in NSW from 1993, as recorded in the NSW Notifiable Conditions Information Management System. For each individual with an HCV notification, records were linked with several administrative data sets: the NSW Admitted Patient Data Collection Database, which includes hospital admissions from 2001; the NSW Registry of Births, Deaths, and Marriages, which includes death registrations from 1993; the NSW Controlled Drugs Data Collection, which includes data on OAT since 1985; and the NSW Bureau of Crime Statistics and Research data set, which includes information about incarceration episodes from 1994. Hepatitis C was chosen as the spine for the study population given that in Australia, (1) it is estimated that >80% of the population of persons who inject drugs have ever had an HCV infection, (2) notification is mandatory, and (3) there is high coverage of screening and diagnosis [17, 18].

### Study Period

Data were extracted from each database as follows: HCV notifications (1 January 1993–31 March 2022), hospitalizations (1 July 2001–31 December 2022), death registrations (1 January 1993–31 December 2022), OAT (1 January 1985–30 November 2022), and incarcerations (1 January 1994–31 December 2021). The study period of interest for outcome analyses spanned 1 July 2001 to 31 December 2022, given availability of hospitalization data.

### Study Cohort and Inclusion Criteria

All people with an HCV notification between 1 January 1993 and 31 March 2022 who were alive at or after July 2001 were considered for analysis. Acknowledging that most people with an HCV notification will have ever injected drugs and that injecting behavior may change over time [19–22], “recent injecting drug use” was defined by hospitalization involving injecting drug use (opioids, stimulants, or other drugs), which was flagged by a previously validated subset of *International Classification of Diseases, Tenth Revision* (*ICD-10*) diagnostic codes occurring within the primary or secondary diagnostic fields (Supplementary Table 1) [23]. Any unplanned inpatient hospitalization occurring 12 months before or after this flag was accepted as occurring within a window of “recent injecting drug use”; this acknowledges the individual trajectories of injecting drug use and that people who inject drugs often seek tertiary health care once their medical complications have become severe [20]. Among these eligible hospitalizations, injecting-related infections were identified in a subset of *ICD-10* codes appearing in primary or secondary diagnostic fields, which were determined from the combination of previous literature and review of the *ICD-10* codebook by the authorship group [20] (Supplementary Table 2). Injecting-related infection hospitalization was considered the index hospitalization for subsequent analysis (Supplementary Figure 1).

### Exclusion Criteria

Several exclusion criteria were applied. First, planned or nonacute hospitalizations were excluded irrespective of injecting-related infection status. Second, unplanned acute hospitalizations were excluded when due to dialysis, same-day or overnight chemotherapy, or involuntary psychiatric admission (entire length of stay; ie, no patient-level autonomy to direct discharge). For analysis of discharge and postdischarge outcomes, hospitalizations were excluded if they involved in-hospital death, discharge to palliative care/hospice service, or transfer to another medical facility or they were missing a discharge type.

### Outcomes

Incidence of injecting-related infection hospitalization was assessed among all hospitalizations associated with recent injecting drug use. Subsequent analyses were conducted with injecting-related infection hospitalizations that ended with clinician- or patient-directed discharge. The type of hospital discharge was assessed, where patient-directed discharge was defined as any hospital separation directed by the patient and coded as the standardized term “against medical advice” in hospital data. Incidence of all-cause unplanned hospital readmission (hereafter, readmission) and all-cause mortality based on data from death registrations (hereafter, mortality) was calculated and censored at 30 and 365 days following index hospitalization discharge for readmission and mortality.

### Exposures

The following were explored as variables of interest for patient-directed discharge, readmission, and mortality: age at hospitalization, sex at birth (male, female), Charlson Comorbidity Index [24, 25], region of residence at the time of hospitalization (defined by the region of NSW local health districts falling into metropolitan, outer metropolitan, or regional/rural areas), recent opioid use (no, yes), recent stimulant use (no, yes), recent other injectable drug use (no, yes), recent alcohol use disorder [26] (no, yes), recent incarceration (no, yes), recent receipt of OAT (no, yes), infection syndrome (skin and soft tissue infection only, other infections ± skin and soft tissue infection), intensive care unit (ICU) admission (no, yes), and—for readmission and mortality outcomes only—length of stay (0–2, 3–7, >7 days) and patient-directed discharge at index hospitalization (no, yes).

Charlson Comorbidity Index was defined as the maximum score recorded through all hospitalization records before index hospitalization (categorized as 0, 1–2, ≥3). Receipt of OAT was defined as any dispensation of methadone or buprenorphine. Supplementary Table 3 presents the *ICD-10* admission codes used to identify hospitalizations occurring due to alcohol use disorder. Incarceration was defined as any time spent in custodial settings. ICU admission was defined as any time spent in the ICU during hospital stay.

For all time-updating variables, recency was defined as an event (eg, stimulant use, OAT dispensation, period of incarceration) occurring 12 months before, after, or at the time of presentation to hospital.

### Statistical Analyses

#### Analysis 1: Incidence of Injecting-Related Infections

First, the annual trends in crude number and incidence of injecting-related infection hospitalizations were plotted. Person-years at risk started 12 months before a hospitalization for injecting drug use and ended 12 months after a hospitalization for injecting drug use or at date of death. Injecting-related infection hospitalizations were assessed as a whole and defined as skin and soft tissue infection only or other invasive infections (with or without skin and soft tissue infection). Incidence was also calculated by categorized infectious syndromes (Supplementary Table 2).

#### Analysis 2: Extent and Predictors of Patient-Directed Discharge

The characteristics of people with injecting-related infection were stratified by discharge type (clinician or patient directed), and incidence of patient-directed discharge was calculated per person-days across characteristics of interest. Time at risk of patient-directed discharge started at the date of admission and ended at the date of discharge or day 90 of hospitalization. The total accumulating hazard of patient-directed discharge was assessed via a Nelson-Aalen cumulative hazard curve. Predictors of patient-directed discharge were assessed with the Prentice-Williams-Peterson extension of the Cox proportional hazards model, which accounts for event order by stratification (ie, event *k* cannot occur until event *k* – 1 has occurred) and allows change in covariate values at subsequent events [27]. For discharge analysis, the gap time approach resets time at risk to zero at each new admission. Robust standard errors were obtained by clustering on individual patients. All exposures were evaluated at the unadjusted level. Individual adjusted models for all variables of interest (as listed in the Exposures section) were adjusted for age at hospitalization, sex at birth, and Charlson Comorbidity Index.

#### Analyses 3 and 4: Extent and Predictors of Unplanned Hospital Readmission and Mortality

The incidence of all-cause readmission at 30 and 365 days was evaluated (calculated per person-days), where the time at risk of readmission started at the date of discharge and ended at the date of readmission, date of death, or date of last follow-up (30 and 365 days postdischarge). The incidence of all-cause mortality at 30 and 365 days following injecting-related infection hospital discharge was evaluated (calculated per person-years), where the time at risk of death started at the date of discharge and ended at the date of death or date of last follow-up (30 and 365 days postdischarge or 31 December 2022). The total accumulating hazard of readmission and mortality was assessed by a Nelson-Aalen cumulative hazard curve and stratified by discharge type and by interaction of discharge type × infection type at index hospitalization. As previously indicated, predictors of readmission and mortality were assessed with the Prentice-Williams-Peterson gap time model and, for mortality, the total time approach, where time at risk resets to zero at each new discharge and continues until death or administrative censoring irrespective of subsequent readmissions. All exposures were evaluated at the unadjusted level. Individual adjusted models for all variables of interest (as listed in Exposures with the addition of length of stay and discharge type at index hospitalization) were adjusted as in analysis 2.

### Correction for Multiple Comparisons

Given the large number of variables and multiple outcomes explored, a Bonferroni correction was applied to analyses 2 to 4 to reduce the chance of a type 1 error. The level of significance for Bonferroni-adjusted hazard estimates was *P* ≤ .005 for patient-directed discharge analysis and *P* ≤ .004 for readmission and mortality analysis.

Data cleaning, management and analyses, and creation of figures were conducted in StataNow/SE 18.5 (StataCorp) and R version 4.4.1.

## RESULTS

### Cohort Derivation and Characteristics

**Figure 1.**
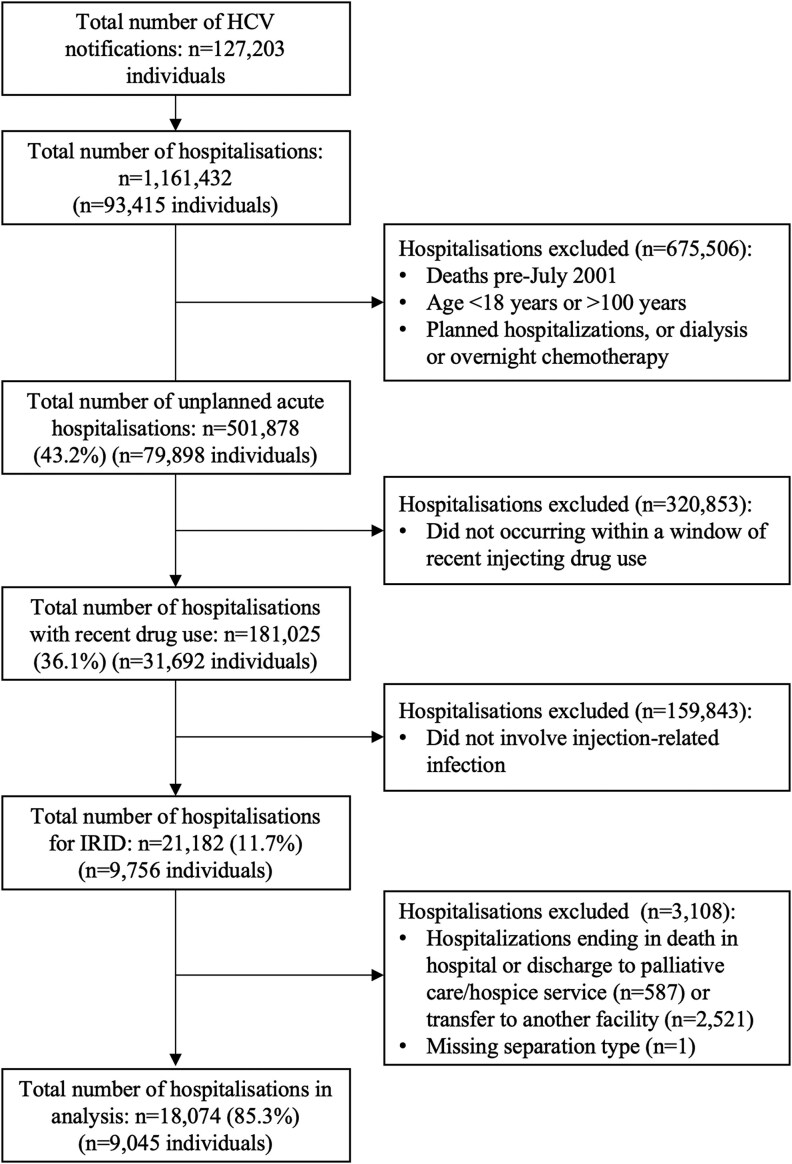
Patient disposition. Abbreviations: HCV, hepatitis C virus; IRID, injecting-related infectious diseases.

Among all people with an HCV notification in NSW between January 1993 and March 2022 who were alive at or after July 2001 (N = 127 203), 79 898 had at least 1 unplanned admission (n = 501 878 hospitalizations). Of these, 31 692 people had an admission with evidence of recent injecting drug use (n = 181 025 hospitalizations), and 9756 had an admission for injecting-related infection (n = 21 182 hospitalizations). Furthermore, 94% of injecting-related infection hospitalizations occurred concurrent with (69%) or in the 12 months after (25%) an injecting drug use hospitalization. Excluding admissions ending in death, transfer to another hospital, or palliative care, 18 074 injecting-related infection hospitalizations were included in the analysis, with a median 2 hospitalizations per person (IQR, 1–3; range, 1–45; Figure 1).

The characteristics of injecting-related infection hospitalizations are presented in Table 1 and Supplementary Figure 1. Median age at hospitalization was 41 years (IQR, 33–49), and most occurred among males (64%). Most admissions were among people who used opioids (n = 13,987, 77%), as compared with those who used stimulants (n = 6723, 37%) or other drugs (n = 5670, 31%). A recent history of alcohol use disorder was evident in 24% of admissions (n = 4428), recent incarceration in 33% (n = 6004), and recent receipt of OAT in 65% (n = 11,675). The median length of stay was 5 days (IQR, 2–12).

**Table 1.** Hospitalizations for Injecting-Related Infection Among People With Recent Injecting Drug Use by Discharge Type

	Injecting-Related Infection Hospitalizations, No. (Column %) or Median (IQR)		
Characteristic^a^	Total	Clinician-Directed Discharge	Patient-Directed Discharge
Admissions	18 074	14 894	3180
Age, y	41 (33–49)	42 (34–50)	39 (32–47)
Sex			
Male	11 548 (64)	9559 (64)	1989 (63)
Female	6523 (36)	5332 (36)	1191 (37)
Missing	3 (<1)	3 (<1)	0 (<1)
Charlson Comorbidity Index			
0	5936 (33)	4895 (33)	1041 (33)
1 or 2	7227 (42)	5879 (39)	1348 (42)
≥3	4911 (25)	4120 (28)	791 (25)
Region of residence at hospitalization			
Metropolitan	5891 (33)	4712 (32)	1179 (37)
Outer metropolitan	5818 (32)	4907 (33)	911 (29)
Regional/rural	6239 (35)	5179 (35)	1061 (33)
Missing	126 (1)	97 (1)	29 (1)
Recent opioid use			
No	4087 (23)	3334 (22)	753 (24)
Yes	13 987 (77)	11 560 (78)	2427 (76)
Recent stimulant use			
No	11 351 (63)	9691 (65)	1660 (52)
Yes	6723 (37)	5203 (35)	1520 (48)
Recent use of other drugs			
No	12 404 (69)	10 351 (70)	2053 (65)
Yes	5670 (31)	4543 (30)	1127 (35)
Recent alcohol use disorder			
No	13 646 (76)	11 237 (76)	2409 (76)
Yes	4428 (24)	3657 (24)	771 (24)
Recent incarceration			
No	12 070 (67)	10 297 (69)	1773 (56)
Yes	6004 (33)	4597 (31)	1407 (44)
Recent opioid agonist therapy			
No	6399 (35)	5251 (35)	1148 (36)
Yes	11 675 (65)	9643 (65)	2032 (64)
Length of stay, d	5 (2–12)	6 (3–14)	3 (1–7)
Duration of hospitalization, d			
≤2	4972 (28)	3557 (24)	1415 (46)
3–7	6353 (35)	5365 (36)	988 (31)
≥8	6749 (37)	5972 (40)	777 (24)
Admission to intensive care unit			
No	16 261 (90)	13 384 (90)	2877 (90)
Yes	1813 (10)	1510 (10)	303 (10)

^a^ *Recent* refers to 12 months before or after index hospitalization.

### Incidence of Injecting-Related Infection Hospitalization

**Figure 2.**
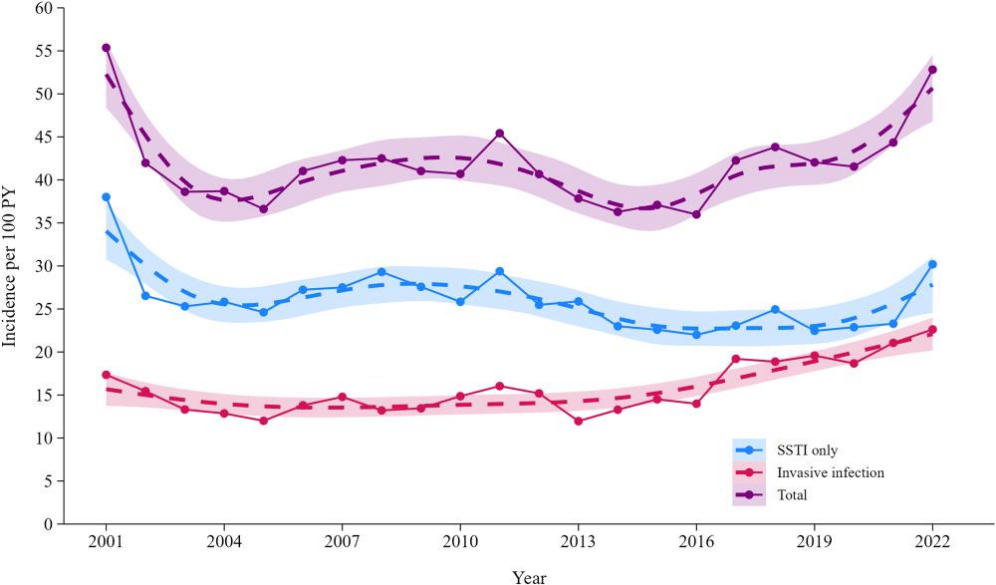
Incidence of hospitalization for injecting-related infection among people with recent injecting drug use, stratified by infection type: invasive infection or skin/soft tissue infection only (SSTI). Data points and lines were smoothed by the generalized additive model method: formula = *y* ∼ *s*(*x*, *bs* = “*cs*”). Shading indicates 95% CI. PY, person-years.

The annual incidence of injecting-related infection hospitalizations is presented in Figure 2 and Supplementary Table 4. Injecting-related infection hospitalization incidence (2001–2022) was 47.2 per 100 person-years. In the last decade (2013–2022), incidence of injecting-related infection hospitalization increased from 37.8 (95% CI, 35.5–40.2) to 52.8 (95% CI, 49.0–56.7) per 100 person-years. While incidence of hospitalization due to skin and soft tissue infections was consistently higher than for invasive infections and was relatively stable over time (25.9 [95% CI, 23.9–27.9] per 100 person-years in 2013 and 30.2 [95% CI, 27.3–33.1] in 2022), invasive infection hospitalization incidence rose from 12.0 (95% CI, 10.6–13.3) per 100 person-years in 2013 to 22.6 (95% CI, 20.2–25.2) in 2022. The median age at hospitalization increased annually (Supplementary Figure 2).

### Patient-Directed Discharge

Among injecting-related infection hospitalizations, 18% (n = 3173) ended with patient-directed discharge, an incidence of 1.6 per 100 person-days (Supplementary Table 5). The incidence of patient-directed discharge was highest in those who were recently incarcerated (2.7 per 100 person-days) and those with recent stimulant use (2.4 per 100 person-days). People hospitalized for skin and soft tissue infection had rates of patient-directed discharge more than double that of people with invasive infections (2.7 and 1.0 per 100 person-days). Supplementary Table 5 presents the incidence of patient-directed discharge by infection type. The median length of stay for a hospitalization that did and did not end in patient-directed discharge was 3 days (IQR, 1–7) and 6 days, respectively [3, 14].

**Figure 3.**
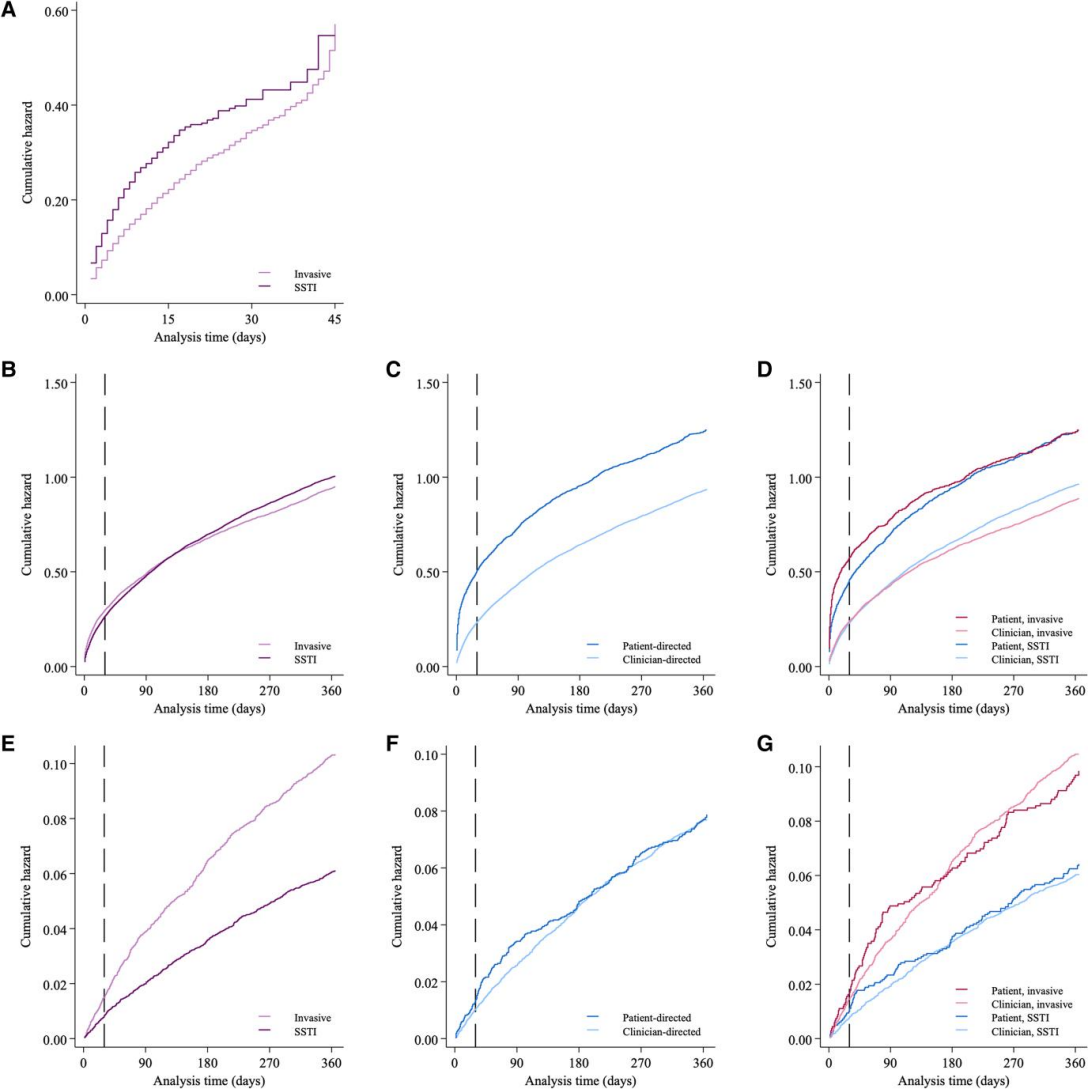
Cumulative hazard of discharge and postdischarge outcomes among people hospitalized with injecting-related infection. *A*, Patient-directed discharge stratified by infection type (invasive infection or skin/soft tissue infection [SSTI] only) at the time of index hospitalization. *B–D*, All-cause unplanned hospital readmission stratified by infection type, discharge status, and interaction of infection type × discharge type. *E–G*, All-cause mortality stratified by infection type, discharge status, and interaction of infection type × discharge type. Dashed line indicates 30 days postdischarge.

**Figure 4.**
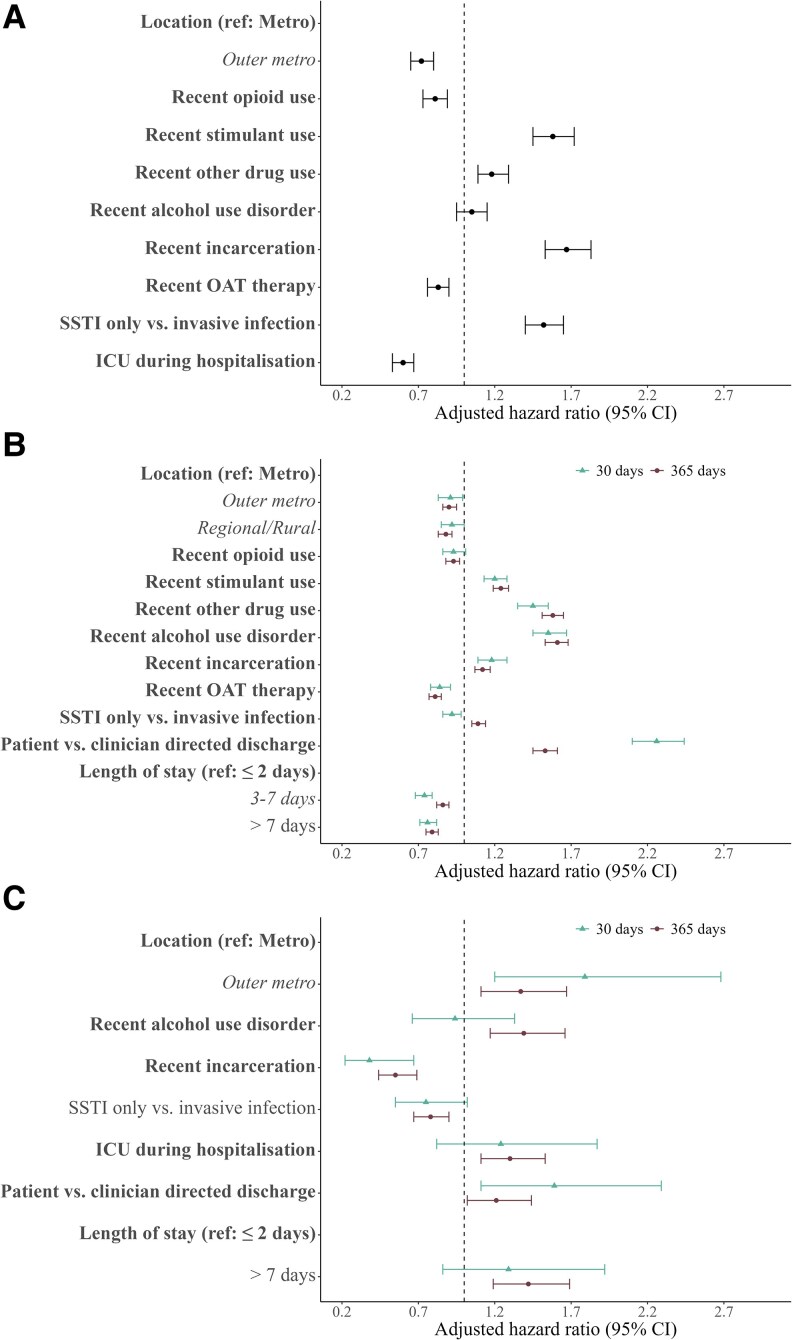
Significant predictors of discharge and posthospital outcomes among people hospitalized with injecting-related infection. *A–C*, Patient-directed discharge, all-cause unplanned hospital readmission, and all-cause mortality. Abbreviations: ICU, intensive care unit; OAT, opiate agonist therapy; SSTI, skin/soft tissue infection.

The cumulative hazard of patient-directed discharge is presented in Figure 3*A*. After adjusting for age, sex, and Charlson Comorbidity Index, correlates associated with patient-directed discharge included recent incarceration (adjusted hazard ratio [aHR], 1.67; 95% CI, 1.53–1.83), recent stimulant use (aHR, 1.58; 95% CI, 1.45–1.72), and hospitalization for skin and soft tissue infection (aHR, 1.52; 95% CI, 1.40–1.65). ICU admission (aHR, 0.60; 95% CI, .53–.67) and recent OAT (aHR, 0.83; 95% CI, .76–.90) were associated with reduced patient-directed discharge (Figure 4*A*, Supplementary Figure 4A, and Supplementary Table 5).

### All-Cause Readmission

There were 4591 (25%) and 10 967 (61%) readmissions at 30 and 365 days, respectively (Supplementary Tables 6 and 7). The incidence of 30-day readmission was 1.0 per 100 person-days and was highest among those with a previous patient-directed discharge (2.05 per 100 person-days). The incidence of 30-day readmission is presented by infection type in Supplementary Table 6.

The cumulative hazard of hospital readmission is presented in Figure 3*B* and 3*C*. After adjusting for age, sex, and Charlson Comorbidity Index, index hospitalization ending in patient-directed discharge was most strongly associated with 30-day readmission (aHR, 2.26; 95% CI, 2.10–2.44). Other correlates associated with higher 30-day readmission were alcohol use disorder (aHR, 1.55; 95% CI, 1.45–1.67), ICU admission (aHR, 1.14; 95% CI, 1.04–1.25), and recent use of stimulants (aHR, 1.20; 95% CI, 1.13–1.28) and other drugs (aHR, 1.45; 95% CI, 1.35–1.55). Correlates that were associated with lower 30-day readmission included recent receipt of OAT (aHR, 0.84; 95% CI, .78–.91) and longer duration of hospitalization vs ≤2 days (3–7 days: aHR, 0.74 [95% CI, .68–.79]; ≥8 days: aHR, 0.76 [95% CI, .71–.82]; Figure 4*B*, Supplementary Figure 4B, and Supplementary Table 6).

The incidence and associations of 365-day readmission are presented in Supplementary Table 7. Correlates of 365-day readmission matched those of 30-day readmission with 2 notable additions: first, skin and soft tissue infection (vs invasive infections) was associated with higher 365-day readmission; second, residence at the time of hospitalization (outer metropolitan or regional/rural vs metropolitan) and recent opioid use were associated with lower 365-day readmission.

### All-Cause Mortality

Following discharge, there were 196 (2%) and 1345 (15%) deaths at 30 and 365 days, respectively (Supplementary Tables 8 and 9). The incidence of 30-day postdischarge mortality was 13.3 per 100 person-years and was highest among those with more severe comorbidities (Charlson Comorbidity Index ≥3; 33.2 per 100 person-years). Incidence of 30-day mortality was higher among those with invasive infections as compared with those with skin and soft tissue infections only (18.2 and 10.2 per 100 person-years). The incidence of 30-day mortality is presented by infection type in Supplementary Table 8.

The cumulative hazard of mortality is presented in Figure 3*D* and 3*E*. After adjusting for age, sex, and Charlson Comorbidity Index, 30-day mortality was associated with region of residence (outer metropolitan vs metropolitan: aHR, 1.79 [95% CI, 1.20–2.68]; regional/rural vs metropolitan: aHR, 1.75 [95% CI, 1.16–2.65]). Recent incarceration was associated with lower 30-day mortality (aHR, 0.38; 95% CI, .22–.67; Supplementary Table 8).

The incidence and associations of 365-day mortality are presented in Supplementary Table 9. There were several additional correlates associated with 365-day mortality, including hospitalization due to recent alcohol use disorder, longer admission duration (≥8 vs ≤2 days), and hospitalizations requiring ICU admission. Hospitalizations involving skin and soft tissue infection only (vs invasive infections) were associated with lower 365-day mortality. Patient-directed discharge was not associated with 30- or 365-day all-cause mortality.

## DISCUSSION

Using a detailed whole-of-population approach, this study provided a comprehensive evaluation of the burden and outcomes of injecting-related infections in NSW. Key findings included increasing injecting-related infection hospitalization incidence and high rates of the adverse outcomes of patient-directed discharge, readmission, and all-cause mortality. Patient-directed discharge occurred in approximately 1 in 5 admissions and was associated with subsequent higher readmission at 1 year, although not all-cause mortality. The study outcomes provide valuable insights into injecting-related infection risk and protective factors—insights that are crucial to strengthening health system and harm reduction responses for people who inject drugs.

Skin and soft tissue injecting-related infection hospitalizations were most common with stable incidence over time, in contrast to hospitalizations for invasive infections, which have increased over the last decade. This rise was postulated to be related to an aging cohort of people who inject drugs in Australia [28, 29]. This is in contrast to the United States, where there is a growing population of young persons who inject drugs [30]. Older persons who inject drugs may be at particular risk of invasive infection given the expected increase in infection susceptibility related to comorbidities, frailty, immunosenescence, and accelerated biological aging [31]. A number of sociodemographic and behavioral factors have been associated with injecting-related infections [32–34]; however, higher-risk behaviors have been typically associated with younger age and shorter duration of injecting drug use [35, 36]. With an aging population, further consideration of the needs of the individual and the health system may be required to optimally manage and prevent infection among older people who inject drugs.

With approximately one-fifth of injecting-related infection hospitalizations ending in patient-directed discharge, this analysis illustrated subpopulations of people who inject drugs with heightened vulnerability (including those with recent stimulant use and recent incarceration) and highlighted areas for health system improvement. Early identification of people at highest risk will be essential to allow for expedited therapeutic interventions, including source control procedures and addiction medicine care. This is underlined by the finding that patient-directed discharge occurred at a median 3 days following admission, which is within the window for drug withdrawal symptoms, which peak at 24 to 48 hours for heroin [37]. People who injected stimulants and other drugs were at higher risk of patient-directed discharge than people who injected opioids, which may reflect the absence of targeted pharmacologic interventions to combat cravings or withdrawal. Previous literature has reported a patient-directed discharge prevalence of 12% to 30% of admissions, with associations with stimulant use, homelessness, HIV, and social/financial temporal pressures [38–41]. While previous literature has been mixed regarding the impact of OAT on patient-directed discharge [42, 43], this analysis reinforced its effectiveness. Similarly, the association with recent incarceration highlighted another particularly vulnerable subpopulation who will require prompt identification and potentially bespoke outpatient models of care, as hospitalization may lead to feelings of loss of autonomy; the period shortly after prison release is a vulnerable time not only for patient-directed discharge but also poor health care engagement and increased mortality [44–48]. It is postulated that this extends to the period before incarceration as well, as it may be a period of increased drug use or risk-taking behaviors with increasing intersection with the justice system [49].

Readmission within 30 and 365 days was significantly more likely following a patient-directed discharge and was postulated to be related to incomplete treatment. Health system improvements and provision of equitable models of care, including access to outpatient antibiotic therapy, could reduce patient-directed discharge and subsequent readmission. Historically, persons who inject drugs were not enrolled into outpatient parenteral antibiotic therapy programs, forcing a decision between a prolonged admission or patient-directed discharge and incomplete treatment. There are several strategies to surmount this: evidence shows that parenteral antibiotic therapy can be delivered safely in selected persons who inject drugs [50–53]; early switch to oral therapy has proven efficacy and may be used, especially when parenteral therapy is not possible [54–58]; and long-acting lipoglycopeptide antibiotics can be administered fortnightly and provide an option for parenteral therapy in those with gram-positive infections [59–62]. In those not medically ready for discharge, prepared plans for patients at risk of sudden patient-directed discharge should be instituted to facilitate ongoing therapy. For example, provision of oral antibiotic therapy at patient-directed discharge is beneficial [63]. With recent OAT therapy being protective against readmission [64], models of care should focus on ensuring longitudinal health care engagement and consider alternative health care settings for infection management.

Among people hospitalized with injecting-related infection, all-cause mortality was associated with infection type and severity, admission duration, alcohol use disorder, and region of residence. As expected, mortality was higher among those who were more comorbid or with severe disease. Alcohol use disorder was associated with higher 365-day mortality, highlighting the contribution of multimorbidity and need to recognize and manage coexisting substance use disorders. Higher mortality was seen among people hospitalized in outer metropolitan and regional/rural areas: this may relate to known general differences in health care access and service provision [65, 66], and differences specific to injecting-related infection could represent a focus for targeted research and intervention. As opposed to readmission, mortality was not associated with patient-directed discharge. This may be a result of those who were able to direct their own discharge being not as unwell and having the mobility and independence to leave, as supported by the association with skin and soft tissue infections vs invasive infections and the negative association with ICU admission. Lower mortality was seen in those with incarceration, which was demonstrated elsewhere [67]. This is thought to be related to institutional supervision and protocols that provide rapid access to care and transfer rather than self-presentation.

The strengths of this study were that it provided longitudinal information on a large cohort of people across many health services. Infections were coded by health systems and did not rely on patient recall. The study's focus on injecting-related infections allowed more detailed analysis of factors associated with adverse outcomes. There were several limitations to this study. Data linkage involved the spine of those with HCV notifications. While some persons who inject drugs will have been excluded, high HCV seroprevalence among persons who inject drugs in Australia will have ensured that most were included and will have increased the specificity for injecting drug use identification. However, persons who inject drugs with and without a history of HCV may represent subpopulations with different risk behavior profiles. With no specific *ICD-10* codes for injecting drug use, identification of people with recent injecting drug use was determined via a set of previously validated codes for other conditions that acted as a surrogate and may have missed cases and included some inappropriately. A further limitation was that the hospitalization data are from NSW only and so could not account for hospitalizations in those who traveled or moved to other states. The study's location in Australia may limit generalizability. For example, among people who inject drugs in Australia, fentanyl use remains low [68], HIV is uncommon [69], and access to harm reduction activities is broad with high uptake [14]. This analysis did not include cause-specific mortality, including infection- and drug-related mortality, which is vital to determine attribution and to design beneficial health strategies; this will be the subject of future work. Beyond data linkage analyses, future research could include quantitative and qualitative exploration of patient, provider, and health system factors that may improve outcomes, such as the impact of addiction medicine interventions beyond OAT; education regarding safe injecting practices [70]; and peer support workers with lived experience to assist with navigation, advocacy, and continuity of care [71, 72].

In conclusion, this whole-of-population analysis conducted over 20 years showed the increasing burden of injecting-related infections in NSW, Australia. Patient-directed discharge was common and was associated with readmission. Detailed analysis of this population has identified individual factors, disease factors, and health system factors that could be targeted with established and novel interventions to improve patient experience, maximize engagement, improve resource utilization, and increase treatment completion.

## Supplementary Material

ofaf257_Supplementary_Data

## Supplementary Data

Supplementary materials are available at *Open Forum Infectious Diseases* online. Consisting of data provided by the authors to benefit the reader, the posted materials are not copyedited and are the sole responsibility of the authors, so questions or comments should be addressed to the corresponding author.

## Notes

***Acknowledgments/patient consent statement.*** This research was produced in whole or part by UNSW Sydney researchers and is subject to the UNSW intellectual property policy. For the purposes of open access, the authors have applied a Creative Commons attribution licence (CC-BY) to any author accepted manuscript version arising from this submission. This study was approved by the NSW Population and Health Services Research Ethics Committee (2019/ETH01777) and the Australian Institute of Health and Welfare Ethics Committee (EO2021/3/1274). Due to the nature of the study, patient consent was waived.

***Author contributions.*** J. M., M. M., B. J., and H. V. were responsible for conceptualization, designs, and methods for the study. B. J., H. V., M. B., and J. M. were responsible for data curation, analysis, and visualization. J. M. wrote the original draft under the supervision of M. M., B. J., and H. V. All authors contributed to critical review and development of the final manuscript for publication.

***Data availability*.** This publication involved information collected by population-based health administration registries. Data used for this research cannot be deposited on servers other than those approved by ethics committees. This publication has used highly sensitive health information through linkage of several administrative data sets. Deidentified linked information has been provided to the research team under strict privacy regulations. Except in the form of conclusions drawn from the data, researchers do not have permission to disclose any data to any person other than those authorized for the research project.

***Funding.*** National Health and Medical Research Council Investigator Grants to G. J. D. (2008276), G. V. M. (2016698), and M. M. (2034418).
