# Supplementary material for: Incidence, Patient-Directed Discharge, Readmission, and Mortality Among People Hospitalized With Injecting-Related Infection: A Population-Based Linkage Study
Source: Open Forum Infect Dis. 2025 Apr 29;12(5):ofaf257. doi: 10.1093/ofid/ofaf257 (PMC12079780; doi:10.1093/ofid/ofaf257)
Supplement: ofaf257_Supplementary_Data [file ofaf257_supplementary_data.docx]

**Supplementary Materials for Incidence, patient-directed discharge, readmission, and mortality among people hospitalized with injecting-related infection: A population-based linkage study**

**Supplementary Table 1:** ICD-10 definitions used to identify injecting drug use-related hospitalizations among all NSW people with an HCV notification

| Drug type | ICD-10 | Code description |
| --- | --- | --- |
| Stimulants | F14 | Mental and behavioural disorders due to use of cocaine |
|  | F15 | Mental and behavioural disorders due to use of other stimulants, including caffeine |
|  | R78.2 | Finding of cocaine in blood |
|  | T40.5 | Poisoning by narcotics and psychodysleptics, cocaine |
|  | T43.6 | Poisoning by psychotropic drugs, not elsewhere classified, psychostimulants with abuse potential |
| Opioids | F11 | Mental and behavioural disorders due to use of opioids |
|  | R78.1 | Finding of opiate drug in blood |
|  | T40.0 | Poisoning by narcotics and psychodysleptics, opium |
|  | T40.1 | Poisoning by narcotics and psychodysleptics, heroin |
|  | T40.2 | Poisoning by narcotics and psychodysleptics, other opioids (codeine/morphine) |
|  | T40.3 | Poisoning by narcotics and psychodysleptics, methadone |
|  | T40.4 | Poisoning by narcotics and psychodysleptics, other synthetic narcotics (pethidine) |
|  | T40.6 | Poisoning by narcotics and psychodysleptics, other and unspecified narcotics |
| Other drugs | F13 | Mental and behavioural disorders due to sedatives or hypnotics |
|  | F19 | Mental and behavioural disorders due to multiple drug use and use of other psychoactive substances |
|  | T38.7 | Androgens and anabolic congeners |
|  | T40.8 | Poisoning by narcotics and psychodysleptics, lysergide (LSD) |
|  | T41.2 | Poisoning by anaesthetics and therapeutic gases, other and unspecified general anaesthetics |
|  | T42.3 | Poisoning by antiepileptic, sedative-hypnotic and antiparkinsonism drugs, barbiturates |
|  | T42.4 | Poisoning by antiepileptic, sedative-hypnotic and antiparkinsonism drugs, benzodiazepines |
|  | T42.5 | Poisoning by antiepileptic, sedative-hypnotic and antiparkinsonism drugs, mixed antiepileptics, not elsewhere classified |
|  | T42.6 | Poisoning by antiepileptic, sedative-hypnotic and antiparkinsonism drugs, other antiepileptic and sedative-hypnotic drugs |
|  | T42.7 | Poisoning by antiepileptic, sedative-hypnotic and antiparkinsonism drugs, antiepileptic and sedative-hypnotic drugs, unspecified |
|  | T42.8 | Poisoning by antiepileptic, sedative-hypnotic and antiparkinsonism drugs, and other central muscle-tone depressants |
|  | T43.8 | Poisoning by psychotropic drugs, not elsewhere classified, other psychotropic drugs, not elsewhere classified |
|  | T43.9 | Poisoning by psychotropic drugs, not elsewhere classified, psychotropic drug, unspecified |
|  | T50.7 | Poisoning by psychotropic drugs, not elsewhere classified, analeptics and opioid receptor antagonists |
|  | X41 | Accidental poisoning by and exposure to antiepileptic, sedative-hypnotic, antiparkinsonism and psychotropic drugs, not elsewhere classified |
|  | X61 | Intentional self-poisoning by and exposure to antiepileptic, sedative-hypnotic, antiparkinsonism and psychotropic drugs, not elsewhere classified |
|  | Y11 | Poisoning by and exposure to antiepileptic, sedative-hypnotic, antiparkinsonism and psychotropic drugs, not elsewhere classified, undetermined intent |

**Supplementary Table 2:** ICD-10 definitions used to identify injecting related infections among all NSW people with an HCV notification

| Infection type | ICD-10 code | Code description |
| --- | --- | --- |
| Bone | M00^*^ | Pyogenic arthritis |
|  | M01.6^*^ | Arthritis in mycoses |
|  | M01.8^*^ | Arthritis in other infectious and parasitic diseases classified elsewhere |
|  | M46.1^*^ | Sacroiliitis, not elsewhere classified |
|  | M46.2^^^ | Osteomyelitis of vertebra |
|  | M46.3 | Infection of intervertebral disc (pyogenic) |
|  | M46.4 | Discitis, unspecified |
|  | M46.5 | Other infective spondylopathies |
|  | M86^#^ | Osteomyelitis |
| Central nervous system | B37.5 | Candidal Meningitis |
|  | B43.1 | Phaeomycotic brain abscess |
|  | G00 | Bacterial meningitis, not elsewhere classified |
|  | G01 | Meningitis in bacterial diseases classified elsewhere |
|  | G02 | Meningitis in other infectious and parasitic diseases classified elsewhere |
|  | G03 | Meningitis due to other and unspecified causes |
|  | G06 | Intracranial and intraspinal abscess and granuloma |
|  | G07 | Intracranial and intraspinal abscess and granuloma in diseases classified elsewhere |
|  | G08 | Intracranial and intraspinal phlebitis and thrombophlebitis |
| Cardiovascular | B37.6 | Candidal Endocarditis |
|  | I33 | Acute and subacute endocarditis |
|  | I38 | Endocarditis, valve unspecified |
|  | I40.0 | Infective myocarditis |
|  | T82.6 | Infection and inflammatory reaction due to cardiac valve prosthesis |
|  | T82.7 | Infection and inflammatory reaction due to other cardiac and vascular devices, implants and grafts |
| Sepsis and/or bloodstream infection | A40 | Streptococcal sepsis |
|  | A41 | Other sepsis |
|  | A42.7 | Actinomycotic sepsis |
|  | A48.3 | Toxic shock syndrome |
|  | B37.7 | Sepsis due to Candida |
|  | R57.2 | Septic shock |
|  | R57.8 | Endotoxic shock NOS |
|  | R65.1 | Severe sepsis |
| Skin and soft tissue infection | A46 | Erysipelas |
|  | A48.0 | Gas gangrene |
|  | B43.2^†^ | Subcutaneous phaeomycotic abscess and cyst |
|  | L00 | Staphylococcal scalded skin syndrome |
|  | L02^†^ | Cutaneous abscess, furuncle, and carbuncle |
|  | L03^β^ | Cellulitis |
|  | L04 | Acute lymphadenitis |
|  | L08.8 | Other specified local infections of skin and subcutaneous tissue |
|  | L08.9 | Local infection of skin and subcutaneous tissue, unspecified |
|  | M60.0 | Infective myositis |
|  | M63.0 | Myositis in bacterial diseases classified elsewhere |
|  | M63.2 | Myositis in other infectious diseases classified elsewhere |
|  | M65.0^†^ | Abscess of tendon sheath |
|  | M65.1 | Other infective (teno)synovitis |
|  | M71.0^†^ | Abscess of bursa |
|  | M71.1 | Other infective bursitis |
|  | M72.6 | Necrotizing fasciitis |
|  | R02 | Gangrene, nos |
| Other | A31.9 | Mycobacterial infection, unspecified |
|  | D73.3 | Abscess of spleen |
|  | E32.1 | Abscess of thymus |
|  | H44.0 | Purulent endophthalmitis |
|  | H44.1 | Other endophthalmitis |
|  | J85 | Abscess of lung and mediastinum |
|  | K63.0 | Other diseases of intestine, abscess of intestine |
|  | K65.1 | Peritoneal abscess |
|  | K68.1 | Retroperitoneal abscess |
|  | K75.0 | Abscess of liver |
|  | K75.1 | Pylephlebitis without liver abscess |
|  | K83.0 | Cholangitis without liver abscess |
|  | N15.1 | Renal and perinephric abscess |
| Unknown | A49.0 | Staphylococcal infection, unspecified site |
|  | A49.1 | Streptococcal and enterococcal infection, unspecified site |
|  | A49.8 | Other bacterial infections of unspecified site |
|  | A49.9 | Bacterial infection, unspecified |
|  | B95 | Streptococcus and staphylococcus as the cause of diseases classified to other chapters |
|  | B96 | Other specified bacterial agents as the cause of diseases classified to other chapters |

^*^ further subcategorised as arthritis

^^^ further subcategorised as vertebral osteomyelitis

^#^ further subcategorised as non-vertebral osteomyelitis

^†^ further subcategorised as abscess

^β^ further subcategorised as cellulitis

**Supplementary Table 3:** ICD-10 definitions used to identify alcohol use disorder among all NSW people with an HCV notification

| Alcohol use disorder | ICD-10 | Code Description |
| --- | --- | --- |
|  | E24.4 | Alcohol-induced pseudo-Cushing syndrome |
|  | F10 | Mental and behavioural disorders due to use of alcohol |
|  | G31.2 | Degeneration of nervous system due to alcohol |
|  | G62.1 | Alcoholic polyneuropathy |
|  | G72.1 | Alcoholic myopathy |
|  | I42.6 | Alcoholic cardiomyopathy |
|  | Z50.2 | Alcoholic rehabilitation |
|  | Z71.4 | Alcohol abuse counselling and surveillance |

**Supplementary Table 4:** Annual incidence of injecting-related hospitalization among people with recent injecting drug use, overall and stratified by infection type (invasive infection vs skin and soft tissue infection only)

| Year | Person years | Event | Syndrome | Incidence | Lower CI | Upper CI |
| --- | --- | --- | --- | --- | --- | --- |
| **2001** | 512.99 | 284 | Total hospitalizations | 55.36 | 48.83 | 61.80 |
|  |  | 195 | Skin and soft tissue infection only | 38.01 | 32.75 | 43.47 |
|  |  | 89 | Invasive infections | 17.35 | 13.84 | 21.05 |
| **2002** | 1353.30 | 568 | Total hospitalizations | 41.97 | 38.57 | 45.44 |
|  |  | 359 | Skin and soft tissue infection only | 26.53 | 23.79 | 29.34 |
|  |  | 209 | Invasive infections | 15.44 | 13.37 | 17.59 |
| **2003** | 1359.50 | 525 | Total hospitalizations | 38.62 | 35.38 | 41.92 |
|  |  | 344 | Skin and soft tissue infection only | 25.30 | 22.66 | 28.03 |
|  |  | 181 | Invasive infections | 13.31 | 11.40 | 15.30 |
| **2004** | 1579.15 | 611 | Total hospitalizations | 38.69 | 35.65 | 41.79 |
|  |  | 408 | Skin and soft tissue infection only | 25.84 | 23.37 | 28.37 |
|  |  | 203 | Invasive infections | 12.85 | 11.15 | 14.63 |
| **2005** | 1807.92 | 662 | Total hospitalizations | 36.62 | 33.85 | 39.44 |
|  |  | 445 | Skin and soft tissue infection only | 24.61 | 22.35 | 26.94 |
|  |  | 217 | Invasive infections | 12.00 | 10.45 | 13.61 |
| **2006** | 1608.48 | 660 | Total hospitalizations | 41.03 | 37.92 | 44.20 |
|  |  | 438 | Skin and soft tissue infection only | 27.23 | 24.68 | 29.78 |
|  |  | 222 | Invasive infections | 13.80 | 12.00 | 15.67 |
| **2007** | 1454.44 | 615 | Total hospitalizations | 42.28 | 38.98 | 45.65 |
|  |  | 400 | Skin and soft tissue infection only | 27.50 | 24.82 | 30.25 |
|  |  | 215 | Invasive infections | 14.78 | 12.86 | 16.78 |
| **2008** | 1529.15 | 650 | Total hospitalizations | 42.51 | 39.30 | 45.78 |
|  |  | 448 | Skin and soft tissue infection only | 29.30 | 26.62 | 32.04 |
|  |  | 202 | Invasive infections | 13.21 | 11.44 | 15.04 |
| **2009** | 1479.31 | 607 | Total hospitalizations | 41.03 | 37.79 | 44.34 |
|  |  | 408 | Skin and soft tissue infection only | 27.58 | 24.94 | 30.28 |
|  |  | 199 | Invasive infections | 13.45 | 11.63 | 15.34 |
| **2010** | 1594.64 | 649 | Total hospitalizations | 40.70 | 37.63 | 43.83 |
|  |  | 412 | Skin and soft tissue infection only | 25.84 | 23.39 | 28.34 |
|  |  | 237 | Invasive infections | 14.86 | 12.98 | 16.81 |
| **2011** | 1521.23 | 691 | Total hospitalizations | 45.42 | 42.07 | 48.84 |
|  |  | 447 | Skin and soft tissue infection only | 29.38 | 26.69 | 32.15 |
|  |  | 244 | Invasive infections | 16.04 | 14.07 | 18.08 |
| **2012** | 1942.41 | 790 | Total hospitalizations | 40.67 | 37.84 | 43.55 |
|  |  | 495 | Skin and soft tissue infection only | 25.48 | 23.27 | 27.75 |
|  |  | 295 | Invasive infections | 15.19 | 13.49 | 16.94 |
| **2013** | 2550.06 | 965 | Total hospitalizations | 37.84 | 35.49 | 40.23 |
|  |  | 660 | Skin and soft tissue infection only | 25.88 | 23.92 | 27.88 |
|  |  | 305 | Invasive infections | 11.96 | 10.63 | 13.33 |
| **2014** | 3009.61 | 1092 | Total hospitalizations | 36.28 | 34.16 | 38.44 |
|  |  | 692 | Skin and soft tissue infection only | 22.99 | 21.30 | 24.72 |
|  |  | 400 | Invasive infections | 13.29 | 11.99 | 14.62 |
| **2015** | 3067.81 | 1138 | Total hospitalizations | 37.09 | 34.94 | 39.28 |
|  |  | 693 | Skin and soft tissue infection only | 22.59 | 20.93 | 24.28 |
|  |  | 445 | Invasive infections | 14.51 | 13.17 | 15.87 |
| **2016** | 3368.65 | 1212 | Total hospitalizations | 35.98 | 33.96 | 38.03 |
|  |  | 741 | Skin and soft tissue infection only | 22.00 | 20.42 | 23.60 |
|  |  | 471 | Invasive infections | 13.98 | 12.74 | 15.26 |
| **2017** | 3031.03 | 1281 | Total hospitalizations | 42.26 | 39.95 | 44.61 |
|  |  | 699 | Skin and soft tissue infection only | 23.06 | 21.38 | 24.78 |
|  |  | 582 | Invasive infections | 19.20 | 17.65 | 20.79 |
| **2018** | 2601.44 | 1140 | Total hospitalizations | 43.82 | 41.28 | 46.40 |
|  |  | 649 | Skin and soft tissue infection only | 24.95 | 23.06 | 26.87 |
|  |  | 491 | Invasive infections | 18.87 | 17.22 | 20.57 |
| **2019** | 2742.72 | 1153 | Total hospitalizations | 42.04 | 39.63 | 44.48 |
|  |  | 616 | Skin and soft tissue infection only | 22.46 | 20.71 | 24.25 |
|  |  | 537 | Invasive infections | 19.58 | 17.94 | 21.26 |
| **2020** | 2683.67 | 1115 | Total hospitalizations | 41.55 | 39.13 | 44.01 |
|  |  | 614 | Skin and soft tissue infection only | 22.88 | 21.09 | 24.70 |
|  |  | 501 | Invasive infections | 18.67 | 17.07 | 20.31 |
| **2021** | 2104.02 | 933 | Total hospitalizations | 44.34 | 41.54 | 47.20 |
|  |  | 490 | Skin and soft tissue infection only | 23.29 | 21.25 | 25.38 |
|  |  | 443 | Invasive infections | 21.05 | 19.11 | 23.05 |
| **2022** | 1387.88 | 733 | Total hospitalizations | 52.81 | 41.54 | 47.20 |
|  |  | 419 | Skin and soft tissue infection only | 30.19 | 27.31 | 33.14 |
|  |  | 314 | Invasive infections | 22.62 | 20.17 | 25.15 |

**
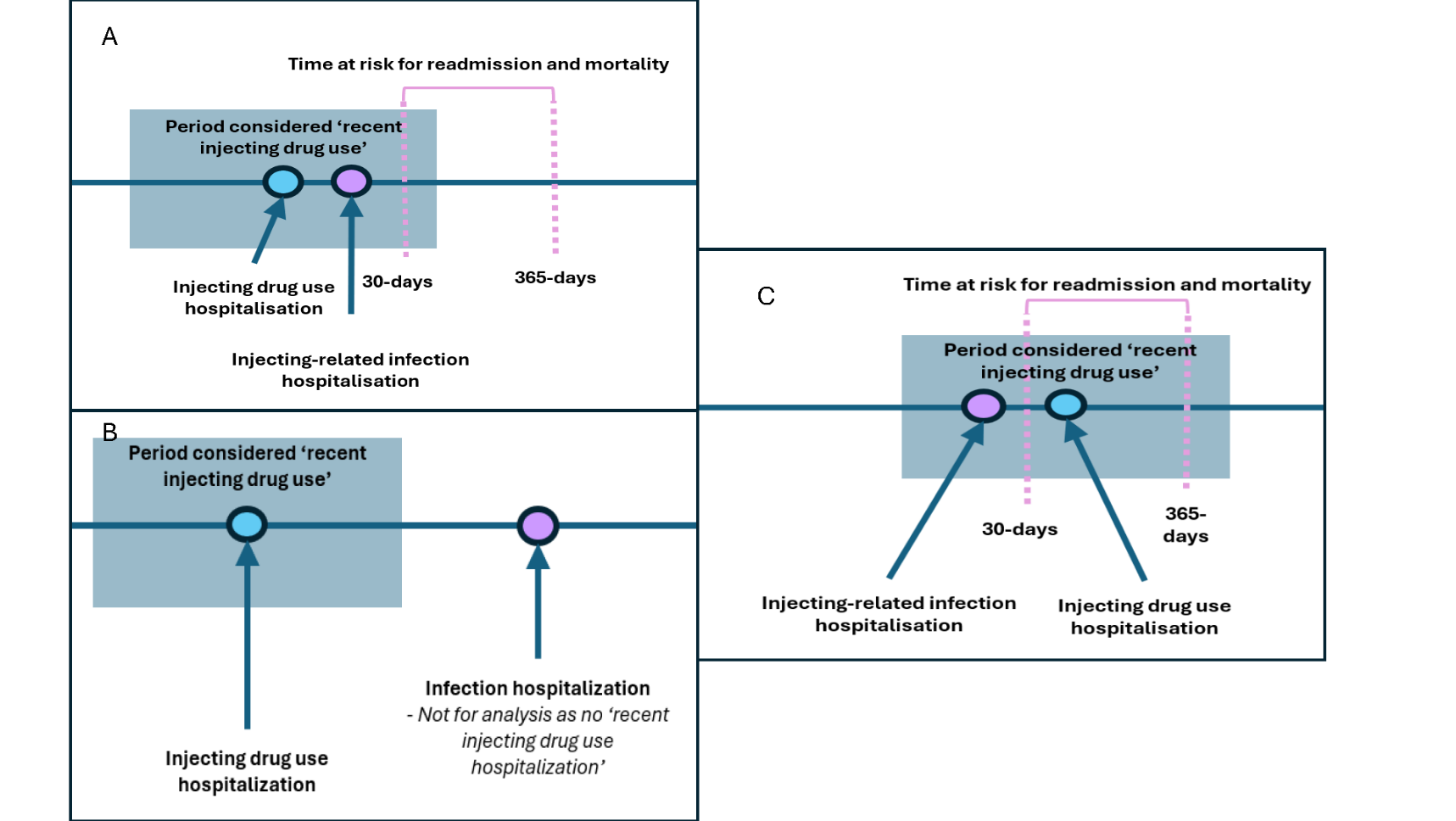
**

**Supplementary Figure 1:** Illustrative timeline of hypothetical participant that would be included for analysis with A) an admission with infection within 12 months after an admission with evidence of injecting, B) admissions with injecting and infection that are more than 12 months apart which would then not be included for analysis, and C) an admission for infection within 12 months prior to an admission with evidence of injecting


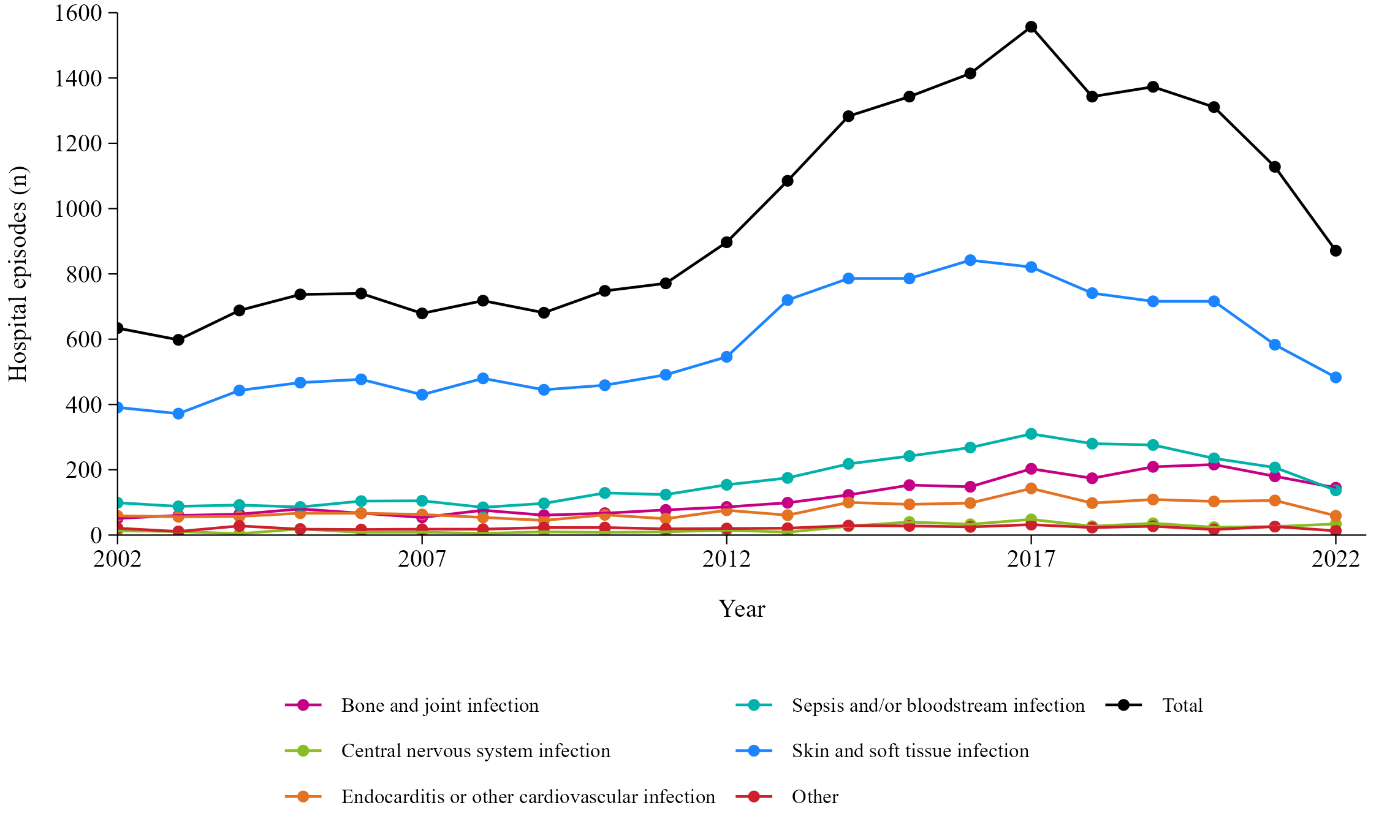
 **Supplementary Figure 2:** Crude number of injecting-related infection hospital diagnoses, by year of admission


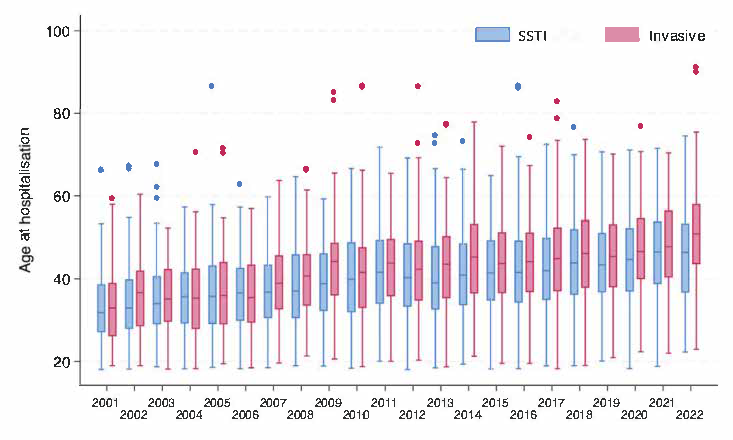


**Supplementary Figure 3:** Distribution of age at hospitalization for each year, stratified by infection type (skin/soft tissue infection only (SSTI) or invasive infection (invasive))


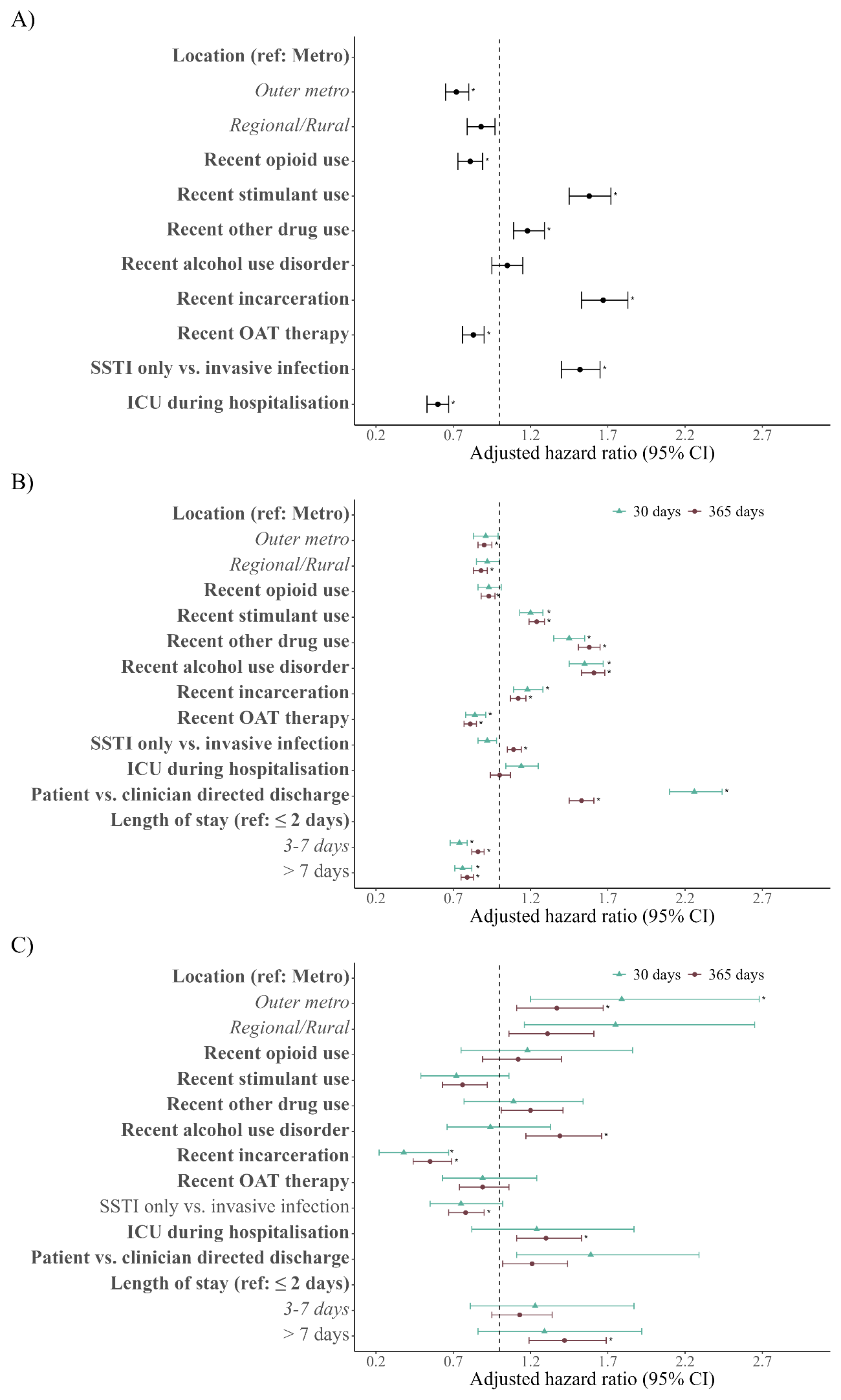


**Supplementary Figure 4**: Predictors of discharge and post-hospital outcomes among people hospitalized with injecting-related infection. Patient-directed discharge (A), all-cause unplanned hospital readmission (B), and all-cause mortality (C). Asterisks (*) denote predictors that are statistically significant following Bonferroni correction for multiple comparisons.

**Supplementary Table 5**: Predictors of patient-directed discharge following injecting-related infection hospitalizations

| Characteristics | | Person-days | Events (n) | Rate (95% CI),  per 100 person-days | HR (95%CI) | aHR (95%CI) |
| --- | --- | --- | --- | --- | --- | --- |
| Total number of admissions | | 198,168 | 3,173 | 1.60 (1.52, 1.69) |  |  |
| Age | Median (IQR) |  |  |  | 0.97 (0.97, 0.97) |  |
| Sex | Male | 123,595 | 1986 | 1.61 (1.51, 1.71) | -reference- |  |
|  | Female | 74,548 | 1187 | 1.59 (1.45, 1.75) | 1.01 (0.92, 1.11) |  |
| Charlson comorbidity index score | 0 | 49,993 | 1039 | 2.08 (1.92, 2.25) | -reference- |  |
|  | 1-2 | 79,244 | 1347 | 1.70 (1.58, 1.83) | 0.87 (0.79, 0.96) |  |
|  | 3+ | 68,931 | 787 | 1.14 (1.02, 1.28) | 0.58 (0.51, 0.66) |  |
| Region of residence at hospitalization | Metropolitan | 66,073 | 1178 | 1.78 (1.63, 1.95) | -reference- | -reference- |
|  | Outer-Metropolitan | 69,669 | 906 | 1.30 (1.19, 1.43) | 0.75 (0.67, 0.83) | 0.72 (0.65, 0.80)* |
|  | Regional/Rural | 61,444 | 1060 | 1.73 (1.59, 1.87) | 0.90 (0.81, 0.99) | 0.88 (0.79, 0.97) |
| Recent opioid use | No | 36,216 | 752 | 2.08 (1.89, 2.28) | -reference- | -reference- |
|  | Yes | 161,952 | 2421 | 1.49 (1.41, 1.59) | 0.78 (0.71, 0.86) | 0.81 (0.73, 0.89)* |
| Recent stimulant use | No | 135,415 | 1653 | 1.22 (1.14, 1.31) | -reference- | -reference- |
|  | Yes | 62,753 | 1520 | 2.42 (2.26, 2.60) | 1.77 (1.63, 1.92) | 1.58 (1.45, 1.72)* |
| Recent use of other drugs | No | 138,525 | 2048 | 1.48 (1.39, 1.57) | -reference- | -reference- |
|  | Yes | 59,643 | 1125 | 1.89 (1.73, 2.06) | 1.24 (1.14, 1.35) | 1.18 (1.09, 1.29)* |
| Recent alcohol use disorder | No | 147,887 | 2405 | 1.63 (1.53, 1.73) | -reference- | -reference- |
|  | Yes | 50,281 | 768 | 1.53 (1.38, 1.70) | 0.94 (0.85, 1.04) | 1.05 (0.95, 1.15) |
| Recent incarceration | No | 146,936 | 1767 | 1.20 (1.12, 1.29) | -reference- | -reference- |
|  | Yes | 51,232 | 1406 | 2.74 (2.55, 2.95) | 2.00 (1.84, 2.17) | 1.67 (1.53, 1.83)* |
| Recent opioid agonist therapy | No | 64,559 | 1146 | 1.78 (1.64, 1.92) | -reference- | -reference- |
|  | Yes | 133,609 | 2027 | 1.52 (1.42, 1.62) | 0.89 (0.81, 0.97) | 0.83 (0.76, 0.90)* |
| Skin and soft tissue infection only | No | 130,118 | 1358 | 1.04 (0.97, 1.12) | -reference- | -reference- |
|  | Yes | 68,050 | 1815 | 2.67 (2.50, 2.85) | 1.58 (1.46, 1.71) | 1.52 (1.40, 1.65)* |
| Admission requiring time in intensive care unit | No | 152,699 | 2875 | 1.88 (1.78, 1.99) | -reference- | -reference- |
|  | Yes | 45,469 | 298 | 0.66 (0.58, 0.75) | 0.54 (0.48, 0.61) | 0.60 (0.53, 0.67)* |
| Infection type |  |  |  |  |  |  |
| Bone and joint infection | All | 53,036 | 444 | 0.84 (0.75, 0.94) |  | |
|  | Septic arthritis | 19,682 | 189 | 0.96 (0.80, 1.15) |  |  |
|  | Vertebral osteomyelitis | 8,376 | 53 | 0.63 (0.47, 0.87) |  |  |
|  | Non-vertebral osteomyelitis | 24,105 | 177 | 0.73 (0.62, 0.88) |  |  |
| Central nervous system infection | All | 12,152 | 74 | 0.61 (0.47, 0.80) |  |  |
| Endocarditis or other cardiovascular infection | All | 39,150 | 362 | 0.92 (0.80, 1.08) |  |  |
| Sepsis and/or bloodstream infection | All | 64,659 | 708 | 1.09 (1.00, 1.20) |  |  |
| Skin and soft tissue | All | 97,665 | 2039 | 2.09 (1.96, 2.22) |  |  |
|  | Abscess | 33,058 | 820 | 2.48 (2.26, 2.72) |  |  |
|  | Cellulitis | 61,415 | 1323 | 2.15 (2.00, 2.32) |  |  |

Note: * denotes statistically significant factors following Bonferroni correction (adjusted alpha: 0.005)

Recent refers to 12 month pre- or post- index hospitalization

**Supplementary Table 6**: Predictors of 30-day hospital readmission following discharge from hospitalization involving injecting related infection

| Characteristics | | Person-days | Events (n) | Rate (95% CI),  per 100 person-days | HR (95%CI) | aHR (95%CI) |
| --- | --- | --- | --- | --- | --- | --- |
| Total number of admissions | | 445,726 | 4,591 | 1.03 (0.99, 1.07) |  |  |
| Age | Median (IQR) |  |  |  | 1.00 (1.00, 1.01) |  |
| Sex | Male | 281,828 | 3,043 | 1.08 (1.03, 1.13) | -reference- |  |
|  | Female | 163,836 | 1,547 | 0.94 (0.88, 1.02) | 0.86 (0.80, 0.93) |  |
| Charlson comorbidity index score | 0 | 152,414 | 1,207 | 0.79 (0.74, 0.85) | -reference- |  |
|  | 1-2 | 181,971 | 1,683 | 0.92 (0.87, 0.99) | 1.08 (0.99, 1.17) |  |
|  | 3+ | 111,342 | 1,701 | 1.53 (1.43, 1.64) | 1.57 (1.43, 1.72) |  |
| Region of residence at hospitalization | Metropolitan | 143,186 | 1,627 | 1.14 (1.06, 1.22) | -reference- | -reference- |
|  | Outer-Metropolitan | 144,663 | 1,415 | 0.98 (0.91, 1.05) | 0.89 (0.82, 0.97) | 0.91 (0.83, 0.99) |
|  | Regional/Rural | 154,684 | 1,519 | 0.98 (0.92, 1.05) | 0.90 (0.83, 0.98) | 0.92 (0.85, 1.00) |
| Recent opioid use | No | 100,665 | 1,037 | 1.03 (0.95, 1.12) | -reference- | -reference- |
|  | Yes | 345,061 | 3,554 | 1.03 (0.98, 1.08) | 0.93 (0.86, 1.00) | 0.93 (0.86, 1.01) |
| Recent stimulant use | No | 283,609 | 2,717 | 0.96 (0.91, 1.01) | -reference- | -reference- |
|  | Yes | 162,117 | 1,874 | 1.16 (1.09, 1.23) | 1.18 (1.10, 1.26) | 1.20 (1.13, 1.28)* |
| Recent use of other drugs | No | 312,848 | 2,768 | 0.88 (0.84, 0.93) | -reference- | -reference- |
|  | Yes | 132,878 | 1,823 | 1.37 (1.29, 1.46) | 1.46 (1.36, 1.57) | 1.45 (1.35, 1.55)* |
| Recent alcohol use disorder | No | 344,968 | 3,030 | 0.88 (0.84, 0.92) | -reference- | -reference- |
|  | Yes | 100,758 | 1,561 | 1.55 (1.44, 1.66) | 1.65 (1.53, 1.77) | 1.55 (1.45, 1.67)* |
| Recent incarceration | No | 298,726 | 3,009 | 1.01 (0.96, 1.06) | -reference- | -reference- |
|  | Yes | 147,000 | 1,582 | 1.08 (1.01, 1.15) | 1.11 (1.04, 1.19) | 1.18 (1.09, 1.28)* |
| Recent opioid agonist therapy | No | 154,463 | 1,765 | 1.14 (1.07, 1.22) | -reference- | -reference- |
|  | Yes | 291,263 | 2,826 | 0.97 (0.92, 1.02) | 0.82 (0.76, 0.88) | 0.84 (0.78, 0.91)* |
| Skin and soft tissue infection only | No | 167,633 | 1,904 | 1.14 (1.08, 1.20) | -reference- | -reference- |
|  | Yes | 278,093 | 2,687 | 0.97 (0.92, 1.02) | 0.88 (0.83, 0.94) | 0.92 (0.86, 0.98) |
| Duration of hospital stay (days) | ≤2 | 117,533 | 1,423 | 1.21 (1.14, 1.29) | -reference- | -reference- |
|  | 3-7 | 161,022 | 1,481 | 0.92 (0.86, 0.98) | 0.77 (0.71, 0.82) | 0.74 (0.68, 0.79)* |
|  | ≥8 | 167,172 | 1,687 | 1.01 (0.96, 1.07) | 0.82 (0.77, 0.88) | 0.76 (0.71, 0.82)* |
| Admission requiring time in intensive care unit | No | 402,878 | 4,066 | 1.01 (0.97, 1.05) | -reference- | -reference- |
|  | Yes | 42,848 | 525 | 1.23 (1.12, 1.35) | 1.23 (1.12, 1.35) | 1.14 (1.04, 1.25) |
| Hospitalisation ending in patient-directed discharge | No | 381,888 | 3,284 | 0.86 (0.82, 0.90) | -reference- | -reference- |
|  | Yes | 63,838 | 1,307 | 2.05 (1.91, 2.20) | 2.19 (2.03, 2.35) | 2.26 (2.10, 2.44)* |
| Infection type |  |  |  |  |  |  |
| Bone and joint infection | All | 57,577 | 668 | 1.16 (1.06, 1.27) |  | |
|  | Septic arthritis | 22,378 | 235 | 1.05 (0.91, 1.22) |  |  |
|  | Vertebral osteomyelitis | 7,308 | 78 | 1.07 (0.84, 1.37) |  |  |
|  | Non-vertebral osteomyelitis | 26,060 | 335 | 1.29 (1.13, 1.47) |  |  |
| Central nervous system infection | All | 10,033 | 108 | 1.08 (0.87, 1.34) |  |  |
| Endocarditis or other cardiovascular infection | All | 38,335 | 493 | 1.29 (1.15, 1.44) |  |  |
| Sepsis and/or bloodstream infection | All | 85,158 | 975 | 1.14 (1.07, 1.23) |  |  |
| Skin and soft tissue | All | 309,865 | 3,051 | 0.98 (0.94, 1.04) |  |  |
|  | Abscess | 121,998 | 1,031 | 0.85 (0.78, 0.92) |  |  |
|  | Cellulitis | 203,776 | 2,069 | 1.02 (0.96, 1.07) |  |  |

Note: * denotes statistically significant factors following Bonferroni correction (adjusted alpha: 0.004)

Recent refers to 12 month pre- or post- index hospitalization

**Supplementary Table 7**: Predictors of 365-day hospital readmission following discharge from hospitalization involving injecting related infection

| Characteristics | | Person-days | Events (n) | Rate (95% CI),  per 100 person-days | HR (95%CI) | aHR (95%CI) |
| --- | --- | --- | --- | --- | --- | --- |
| Total number of admissions | | 3,357,322 | 10,967 | 0.33 (0.32, 0.34) |  |  |
| Age | Median (IQR) |  |  |  | 1.00 (1.00, 1.00) |  |
| Sex | Male | 2,111,755 | 6,991 | 0.33 (0.32, 0.34) | -reference- |  |
|  | Female | 1,245,310 | 3,973 | 0.32 (0.30, 0.34) | 0.95 (0.91, 0.99) |  |
| Charlson comorbidity index score | 0 | 1,272,656 | 3,298 | 0.26 (0.25, 0.27) | -reference- |  |
|  | 1-2 | 1,433,934 | 4,235 | 0.30 (0.28, 0.31) | 1.03 (0.97, 1.08) |  |
|  | 3+ | 650,732 | 3,434 | 0.53 (0.50, 0.56) | 1.45 (1.37, 1.54) |  |
| Region of residence at hospitalization | Metropolitan | 1,024,677 | 3,812 | 0.37 (0.35, 0.39) | -reference- | -reference- |
|  | Outer-Metropolitan | 1,101,920 | 3,478 | 0.32 (0.30, 0.33) | 0.89 (0.85, 0.94) | 0.90 (0.86, 0.95)* |
|  | Regional/Rural | 1,207,766 | 3,601 | 0.30 (0.28, 0.31) | 0.86 (0.82, 0.91) | 0.88 (0.83, 0.92)* |
| Recent opioid use | No | 767,033 | 2,505 | 0.33 (0.31, 0.35) | -reference- | -reference- |
|  | Yes | 2,590,289 | 8,462 | 0.33 (0.32, 0.34) | 0.93 (0.88, 0.97) | 0.93 (0.88, 0.97) |
| Recent stimulant use | No | 2,199,020 | 6,516 | 0.30 (0.29, 0.31) | -reference- | -reference- |
|  | Yes | 1,158,302 | 4,451 | 0.38 (0.37, 0.40) | 1.23 (1.18, 1.28) | 1.24 (1.19, 1.29)* |
| Recent use of other drugs | No | 2,531,043 | 6,837 | 0.27 (0.26, 0.28) | -reference- | -reference- |
|  | Yes | 826,279 | 4,130 | 0.50 (0.48, 0.52) | 1.60 (1.53, 1.67) | 1.58 (1.51, 1.65)* |
| Recent alcohol use disorder | No | 2,763,735 | 7,699 | 0.28 (0.27, 0.29) | -reference- | -reference- |
|  | Yes | 593,587 | 3,268 | 0.55 (0.52, 0.58) | 1.67 (1.59, 1.75) | 1.61 (1.53, 1.68)* |
| Recent incarceration | No | 2,237,219 | 7,204 | 0.32 (0.31, 0.34) | -reference- | -reference- |
|  | Yes | 1,120,103 | 3,763 | 0.34 (0.32, 0.35) | 1.08 (1.04, 1.13) | 1.12 (1.07, 1.17)* |
| Recent opioid agonist therapy | No | 1,095,481 | 4,109 | 0.38 (0.36, 0.39) | -reference- | -reference- |
|  | Yes | 2,261,841 | 6,858 | 0.30 (0.29, 0.32) | 0.81 (0.77, 0.84) | 0.81 (0.77, 0.85)* |
| Skin and soft tissue infection only | No | 1,283,109 | 4,114 | 0.32 (0.31, 0.34) | -reference- | -reference- |
|  | Yes | 2,074,213 | 6,853 | 0.33 (0.32, 0.34) | 1.05 (1.01, 1.09) | 1.09 (1.05, 1.14)* |
| Duration of hospital stay (days) | ≤2 | 884,570 | 3,194 | 0.36 (0.34, 0.38) | -reference- | -reference- |
|  | 3-7 | 1,207,088 | 3,867 | 0.32 (0.31, 0.34) | 0.88 (0.84, 0.92) | 0.86 (0.82, 0.90)* |
|  | ≥8 | 1,265,664 | 3,906 | 0.31 (0.30, 0.32) | 0.84 (0.80, 0.88) | 0.79 (0.75, 0.83)* |
| Admission requiring time in intensive care unit | No | 3,041,021 | 9,880 | 0.32 (0.31, 0.34) | -reference- | -reference- |
|  | Yes | 316,301 | 1,087 | 0.34 (0.32, 0.37) | 1.08 (1.01, 1.15) | 1.00 (0.94, 1.07) |
| Hospitalisation ending in patient-directed discharge | No | 2,902,048 | 8,748 | 0.30 (0.29, 0.31) | -reference- | -reference- |
|  | Yes | 455,274 | 2,219 | 0.49 (0.46, 0.52) | 1.50 (1.42, 1.59) | 1.53 (1.45, 1.61)* |
| Infection type |  |  |  |  |  |  |
| Bone and joint infection | All | 454,857 | 1,429 | 0.31 (0.29, 0.34) |  | |
|  | Septic arthritis | 183,899 | 537 | 0.29 (0.26, 0.32) |  |  |
|  | Vertebral osteomyelitis | 65,254 | 161 | 0.25 (0.21, 0.30) |  |  |
|  | Non-vertebral osteomyelitis | 186,203 | 710 | 0.38 (0.34, 0.43) |  |  |
| Central nervous system infection | All | 86,615 | 220 | 0.25 (0.21, 0.30) |  |  |
| Endocarditis or other cardiovascular infection | All | 295,736 | 982 | 0.33 (0.30, 0.36) |  |  |
| Sepsis and/or bloodstream infection | All | 632,249 | 2,096 | 0.33 (0.31, 0.35) |  |  |
| Skin and soft tissue | All | 2,309,406 | 7,676 | 0.33 (0.32, 0.34) |  |  |
|  | Abscess | 944,263 | 2,893 | 0.31 (0.29, 0.32) |  |  |
|  | Cellulitis | 1,503,809 | 5,082 | 0.34 (0.32, 0.35) |  |  |

Note: * denotes statistically significant factors following Bonferroni correction (adjusted alpha: 0.004)

Recent refers to 12 month pre- or post- index hospitalization

**Supplementary Table 8**: Predictors of 30-day all-cause mortality following discharge from hospitalization involving injecting related infection

| Characteristics | | Person-years | Events (n) | Rate (95% CI),  per 100PY | HR (95%CI) | aHR (95%CI) |
| --- | --- | --- | --- | --- | --- | --- |
| Total number of admissions | | 1,476 | 196 | 13.28 (11.54, 15.27) |  |  |
| Age |  |  |  |  | 1.07 (1.06, 1.09) |  |
| Sex | Male | 942 | 139 | 14.75 (12.25, 17.91) | -reference- |  |
|  | Female | 534 | 57 | 10.68 (8.10, 14.39) | 0.70 (0.50, 0.99) |  |
| Charlson comorbidity index score | 0 | 487 | 23 | 4.73 (3.03, 7.80) | -reference- |  |
|  | 1-2 | 592 | 41 | 6.93 (5.04, 9.79) | 1.35 (0.75, 2.42) |  |
|  | 3+ | 398 | 132 | 33.18 (27.39, 40.56) | 6.07 (3.53,10.46) |  |
| Region of residence at hospitalization | Metropolitan | 482 | 47 | 9.76 (7.21, 13.56) | -reference- | -reference- |
|  | Outer-Metropolitan | 475 | 72 | 15.16 (11.86, 19.69) | 1.63 (1.10, 2.42) | 1.79 (1.20, 2.68)* |
|  | Regional/Rural | 509 | 76 | 14.92 (11.54, 19.64) | 1.64 (1.09, 2.47) | 1.75 (1.16, 2.65) |
| Recent opioid use | No | 334 | 33 | 9.88 (6.58, 15.52) | -reference- | -reference- |
|  | Yes | 1,142 | 163 | 14.27 (12.08, 16.99) | 1.28 (0.81, 2.02) | 1.18 (0.75, 1.86) |
| Recent stimulant use | No | 926 | 147 | 15.87 (13.31, 19.07) | -reference- | -reference- |
|  | Yes | 550 | 49 | 8.91 (6.45, 12.67) | 0.55 (0.37, 0.80) | 0.72 (0.49, 1.06) |
| Recent use of other drugs | No | 1,013 | 129 | 12.73 (10.57, 15.48) | -reference- | -reference- |
|  | Yes | 463 | 67 | 14.47 (11.00, 19.45) | 1.07 (0.76, 1.51) | 1.09 (0.77, 1.54) |
| Recent alcohol use disorder | No | 1,115 | 135 | 12.11 (10.06, 14.71) | -reference- | -reference- |
|  | Yes | 361 | 61 | 16.89 (12.80, 22.74) | 1.33 (0.94, 1.88) | 0.94 (0.66, 1.33) |
| Recent incarceration | No | 984 | 177 | 17.99 (15.32, 21.27) | -reference- | -reference- |
|  | Yes | 492 | 19 | 3.86 (2.39, 6.66) | 0.23 (0.13, 0.38) | 0.38 (0.22, 0.67)* |
| Recent opioid agonist therapy | No | 522 | 84 | 16.09 (12.71, 20.68) | -reference- | -reference- |
|  | Yes | 954 | 112 | 11.74 (9.57, 14.56) | 0.68 (0.49, 0.94) | 0.89 (0.63, 1.24) |
| Skin and soft tissue infection only | No | 571 | 104 | 18.23 (14.91, 22.52) | -reference- | -reference- |
|  | Yes | 906 | 92 | 10.16 (8.08, 12.95) | 0.58 (0.43, 0.80) | 0.75 (0.55, 1.02) |
| Duration of hospital stay (days) | ≤2 | 407 | 33 | 8.11 (5.77, 11.77) | -reference- |  |
|  | 3-7 | 519 | 68 | 13.10 (10.08, 17.37) | 1.61 (1.06, 2.45) | 1.23 (0.81, 1.87) |
|  | ≥8 | 550 | 95 | 17.26 (14.17, 21.25) | 2.06 (1.38, 3.07) | 1.29 (0.86, 1.92) |
| Admission requiring time in intensive care unit | No | 1,329 | 164 | 12.34 (10.40, 14.76) | -reference- | -reference- |
|  | Yes | 147 | 32 | 21.70 (15.36, 31.67) | 1.81 (1.22, 2.69) | 1.24 (0.82, 1.87) |
| Hospitalisation ending in patient-directed discharge | No | 1,217 | 153 | 12.57 (10.64, 14.96) | -reference- |  |
|  | Yes | 259 | 43 | 16.58 (11.84, 23.98) | 1.24 (0.86, 1.79) | 1.59 (1.11, 2.29) |
| Infection type |  |  |  |  |  | |
| Bone and joint infection | All | 198 | 16 | 8.09 (4.91, 14.35) |  | |
|  | Septic arthritis | 76 | 5 | 6.60 (2.26, 27.58) |  |  |
|  | Vertebral osteomyelitis | 25 | 3 | 12.05 (3.76, 59.10) |  |  |
|  | Non-vertebral osteomyelitis | 91 | 9 | 9.94 (5.32, 20.86) |  |  |
| Central nervous system infection | All | 34 | 4 | 11.68 (4.39, 41.87) |  |  |
| Endocarditis or other cardiovascular infection | All | 135 | 28 | 20.80 (14.24, 31.64) |  |  |
| Sepsis and/or bloodstream infection | All | 289 | 75 | 25.92 (20.58, 33.12) |  |  |
| Skin and soft tissue | All | 1,014 | 109 | 10.75 (8.74, 13.39) |  |  |
|  | Abscess | 390 | 16 | 4.11 (2.57, 7.01) |  |  |
|  | Cellulitis | 670 | 94 | 14.03 (11.18, 17.87) |  |  |

Note: * denotes statistically significant factors following Bonferroni correction (adjusted alpha: 0.004)

Recent refers to 12 month pre- or post- index hospitalization

**Supplementary Table 9**: Predictors of 365-day all-cause mortality following discharge from hospitalization involving injecting-related infection

| Characteristics | | Person-years | Events (n) | Rate (95% CI),  per 100PY | HR (95%CI) | aHR (95%CI) |
| --- | --- | --- | --- | --- | --- | --- |
| Total number of admissions | | 17,274 | 1,345 | 7.79 (7.19, 8.44) |  |  |
| Age | Median (IQR) |  |  |  | 1.07 (1.06, 1.07) |  |
| Sex | Male | 10,996 | 921 | 8.38 (7.61, 9.24) | -reference- |  |
|  | Female | 6,275 | 424 | 6.76 (5.88, 7.81) | 0.78 (0.65, 0.93) |  |
| Charlson comorbidity index score | 0 | 5,845 | 157 | 2.69 (2.17, 3.37) | -reference- |  |
|  | 1-2 | 7,028 | 348 | 4.95 (4.31, 5.72) | 1.75 (1.35, 2.29) |  |
|  | 3+ | 4,401 | 840 | 19.09 (17.18, 21.25) | 6.38 (4.93, 8.26) |  |
| Region of residence at hospitalization | Metropolitan | 5,664 | 398 | 7.03 (6.10, 8.14) | -reference- | -reference- |
|  | Outer-Metropolitan | 5,543 | 461 | 8.32 (7.28, 9.54) | 1.23 (1.01, 1.50) | 1.37 (1.11, 1.67)* |
|  | Regional/Rural | 5,945 | 480 | 8.07 (7.07, 9.26) | 1.22 (1.00, 1.49) | 1.31 (1.06, 1.61) |
| Recent opioid use | No | 3,947 | 240 | 6.08 (4.95, 7.56) | -reference- | -reference- |
|  | Yes | 13,327 | 1105 | 8.29 (7.62, 9.04) | 1.22 (0.97, 1.54) | 1.12 (0.89, 1.40) |
| Recent stimulant use | No | 10,763 | 985 | 9.15 (8.36, 10.04) | -reference- | -reference- |
|  | Yes | 6,511 | 360 | 5.53 (4.69, 6.56) | 0.59 (0.49, 0.71) | 0.76 (0.63, 0.92) |
| Recent use of other drugs | No | 11,881 | 862 | 7.26 (6.62, 7.97) | -reference- | -reference- |
|  | Yes | 5,393 | 483 | 8.96 (7.77, 10.38) | 1.17 (0.99, 1.38) | 1.20 (1.01, 1.41) |
| Recent alcohol use disorder | No | 13,150 | 829 | 6.30 (5.73, 6.95) | -reference- | -reference- |
|  | Yes | 4,124 | 516 | 12.51 (10.90, 14.43) | 1.88 (1.58, 2.23) | 1.39 (1.17, 1.66)* |
| Recent incarceration | No | 11,375 | 1,157 | 10.17 (9.33, 11.10) | -reference- | -reference- |
|  | Yes | 5,899 | 188 | 3.19 (2.64, 3.89) | 0.33 (0.27, 0.41) | 0.55 (0.44, 0.69)* |
| Recent opioid agonist therapy | No | 6,060 | 562 | 9.27 (8.18, 10.56) | reference- | -reference- |
|  | Yes | 11,214 | 783 | 6.98 (6.31, 7.75) | 0.71 (0.60, 0.83) | 0.89 (0.74, 1.06) |
| Skin and soft tissue infection only | No | 6,585 | 688 | 10.45 (9.45, 11.57) | -reference- | -reference- |
|  | Yes | 10,689 | 657 | 6.15 (5.49, 6.91) | 0.61 (0.53, 0.71) | 0.78 (0.67, 0.90)* |
| Duration of hospital stay (days) | ≤2 | 4,839 | 233 | 4.82 (4.09, 5.71) | -reference- | -reference- |
|  | 3-7 | 6,098 | 427 | 7.00 (6.24, 7.89) | 1.44 (1.21, 1.72) | 1.13 (0.95, 1.34) |
|  | ≥8 | 6,337 | 685 | 10.81 (9.87, 11.86) | 2.18 (1.83, 2.59) | 1.42 (1.19, 1.69)* |
| Admission requiring time in intensive care unit | No | 15,592 | 1,123 | 7.20 (6.60, 7.87) | -reference- | -reference- |
|  | Yes | 1,682 | 222 | 13.20 (11.47, 15.26) | 1.90 (1.63, 2.21) | 1.30 (1.11, 1.53)* |
| Hospitalisation ending in patient-directed discharge | No | 14,241 | 1,104 | 7.75 (7.14, 8.43) | -reference- | -reference- |
|  | Yes | 3,033 | 241 | 7.95 (6.71, 9.48) | 0.97 (0.81, 1.15) | 1.21 (1.02, 1.44) |
| Infection type |  |  |  |  |  | |
| Bone and joint infection | All | 2,337 | 141 | 6.03 (4.78, 7.73) |  | |
|  | Septic arthritis | 904 | 36 | 3.98 (2.85, 5.73) |  |  |
|  | Vertebral osteomyelitis | 299 | 8 | 2.67 (1.35, 6.02) |  |  |
|  | Non-vertebral osteomyelitis | 1,059 | 91 | 8.59 (6.26, 12.11) |  |  |
| Central nervous system infection | All | 409 | 15 | 3.67 (2.16, 6.72) |  |  |
| Endocarditis or other cardiovascular infection | All | 1,546 | 176 | 11.38 (9.33, 14.03) |  |  |
| Sepsis and/or bloodstream infection | All | 3,278 | 456 | 13.91 (12.43, 15.62) |  |  |
| Skin and soft tissue | All | 11,937 | 785 | 6.58 (5.93, 7.31) |  |  |
|  | Abscess | 4,669 | 149 | 3.19 (2.59, 3.97) |  |  |
|  | Cellulitis | 7,833 | 622 | 7.94 (7.10, 8.91) |  |  |

Note: * denotes statistically significant factors following Bonferroni correction (adjusted alpha: 0.004)

Recent refers to 12 month pre- or post- index hospitalization
